# Muscarinic M1 receptors modulate endotoxemia-induced loss of synaptic plasticity

**DOI:** 10.1186/s40478-015-0245-8

**Published:** 2015-11-04

**Authors:** Aleksandar R. Zivkovic, Oliver Sedlaczek, Rebecca von Haken, Karsten Schmidt, Thorsten Brenner, Markus A. Weigand, Hilmar Bading, C. Peter Bengtson, Stefan Hofer

**Affiliations:** Department of Anesthesiology, Heidelberg University Hospital, Im Neuenheimer Feld 110, 69120 Heidelberg, Germany; Neurobiology, Interdisciplinary Centre for Neurosciences (IZN), University of Heidelberg, Im Neuenheimer Feld 364, 69120 Heidelberg, Germany; Department of Radiology, Heidelberg University Hospital, Im Neuenheimer Feld 110, 69120 Heidelberg, Germany

**Keywords:** Hippocampus, Encephalitis, Neurology, NMDA, Critical illness, Sepsis diagnostics

## Abstract

Septic encephalopathy is associated with rapid deterioration of cortical functions. Using magnetic resonance imaging (MRI) we detected functional abnormalities in the hippocampal formation of patients with septic delirium. Hippocampal dysfunction was further investigated in an animal model for sepsis using lipopolysaccharide (LPS) injections to induce endotoxemia in rats, followed by electrophysiological recordings in brain slices. Endotoxemia induced a deficit in long term potentiation which was completely reversed by apamin, a blocker of small conductance calcium-activated potassium (SK) channels, and partly restored by treatment with physostigmine (eserine), an acetylcholinesterase inhibitor, or TBPB, a selective M1 muscarinic acetylcholine receptor agonist. These results suggest a novel role for SK channels in the etiology of endotoxemia and explain why boosting cholinergic function restores deficits in synaptic plasticity. Drugs which enhance cholinergic or M1 activity in the brain may prove beneficial in treatment of septic delirium in the intensive care unit.

**Electronic supplementary material:**

The online version of this article (doi:10.1186/s40478-015-0245-8) contains supplementary material, which is available to authorized users.

## Introduction

Septic encephalopathy is sepsis-related brain dysfunction with a rapid deterioration of cortical functions leading to disorientation, confusion, cognitive deficits and memory impairment [10, 39, 59]. The pathogenesis is multifactorial and the underlying mechanisms are not yet fully understood [9, 22, 30, 44, 47, 49]. Indeed, septic encephalopathy remains a clinical diagnosis of exclusion with a high mortality rate (50–70 %) [5, 18]. Several clinical screening methods have been developed to improve the diagnosis of septic delirium [4, 21, 58], however pharmacological sedation of many intensive care unit (ICU) patients complicates or prevents the evaluation of their cognitive status [20]. A few novel diagnostic procedures have been recently described and several studies have detected changes in the septic brain using functional brain imaging [3, 23, 42, 45, 53]. However, the accurate diagnosis of septic delirium remains problematic, often requiring the combination of various approaches.

A cholinergic anti-inflammatory pathway is known to modulate the systemic inflammatory response to endotoxin. Inflammatory activation of acetylcholine release from vagus fibres stimulates hypothalamic-pituitary-adrenal axis responses. Vagal stimulation also can attenuate pro-inflammatory cytokine release and alterations in synaptic function accompanying sepsis in the prefrontal cortex [11, 24]. Moreover, advances in the field of neuroimmunology have shown that the nervous and immune systems interact during inflammation [31, 40, 43, 46, 54, 57]. This interaction has been previously described as the ‘inflammatory reflex’ [55]. Our group has previously shown that increasing acetylcholine receptor activity with either blood–brain barrier permeable (physostigmine) or impermeable (neostigmine) cholinesterase inhibitors, which prolong the lifetime of endogenous acetylcholine, reduces intracellular cytokine synthesis by macrophages, thus reducing the inflammatory response and improving survival [26]. However, the alterations in cholinergic neurotransmission or the clinical efficacy of anticholinesterases in sepsis-induced delirium remain unclear.

A causal link between cholinergic dysfunction and delirium has been hypothesized on the basis of clinical observations [27]. Delirium involving impaired learning and memory occurs in central anticholinergic syndrome (CAS), a condition caused by general anesthesia including anticholinergic agents. CAS is commonly treated with cholinesterase inhibitors [32]. Clinical studies examining the treatment of sepsis-induced delirium with the cholinesterase inhibitor, rivastigmine, have shown positive results, although sample sizes were too small to be conclusive [36, 38]. A multi-center double-blind and placebo-controlled study was interrupted due to increased mortality in the rivastigmine treatment group [19]. Thus the use of cholinesterase inhibitors to treat delirium in critically ill patients remains controversial and further studies investigating the pathophysiological mechanism underlying septic delirium are required.

The mechanism linking cholinergic dysfunction with delirium may involve cholinergic modulation of glutamatergic synapses in the hippocampus. Glutamatergic synapses can undergo use-dependent changes in synaptic strength, commonly referred to as synaptic plasticity, believed to underlie learning and memory. Bursts of synaptic activity can induce long lasting strengthening of excitatory synapses, a phenomenon termed ‘long-term potentiation’ (LTP), which was discovered in the hippocampus [7] and has been observed at virtually every glutamatergic synapse in the brain [6]. Interestingly, LTP can be modulated by other neurotransmitters such as dopamine and acetylcholine [37, 56] and the muscarinic M1 subtype of cholinergic receptors are known to mediate synaptic plasticity by inhibiting small-conductance Ca^2+^-activated potassium (SK) channels at hippocampal synapses [8, 13, 25].

Here we present evidence from magnetic resonance imaging (MRI) revealing functional abnormalities in the hippocampus of critically ill patients diagnosed with sepsis-induced encephalopathy. We used a rat model of sepsis, endotoxemia, to investigate alterations in hippocampal function and LTP in rat brain slices using the single cell electrophysiology technique of whole-cell patch clamp. Basic cell functions were not affected by endotoxemia, however, synaptic plasticity was impaired as has been previously shown [33, 52]. Here we identify the involvement of SK channels in this LTP deficit which could be partly rescued by boosting cholinergic function. We thus postulate that SK channels are the mechanistic link explaining why endotoxemia-induced downregulation of cholinergic activity affects hippocampal-dependent cognitive functions by suppressing the ability of hippocampal synapses to undergo changes in their synaptic efficacy.

## Materials and methods

### Patient recruitment criteria

The study included 5 patients diagnosed with severe systemic inflammation, according to the criteria of the Surviving Sepsis Campaign: International Guidelines for Management of Severe Sepsis and Septic Shock [17]. Identifying the precise starting time point of the systemic inflammation in critically ill patients is a complex procedure. Therefore, the inclusion criteria for the patients, based on International Guidelines for Management of Severe Sepsis and Septic Shock allowed for a standardized and early diagnosis and therapy in the ICU environment.

### MRI imaging

MRI was performed on human patients using a 1.5 T scanner (Magnetom Aera; Siemens Medical Systems, Erlangen, Germany) with echo planar hardware (gradient power 45 mT/m and rise time 200 mT/m/ms). After using orthogonal localizers, standard transverse continuous 5 mm images, T2 Flair with a FOV of 270 mm, DWI (echo planar, SE, repetition time 6300 ms; echo time 113 ms, 250 mm FOV, 5 mm slice thickness, 192 × 192 matrix, three b values = 0 to 1000, diffusion gradients in three orthogonal planes) images in the transverse oblique plane were acquired. Further DW sequences and matching high-resolution T2 images (voxel size 0.6 × 0.6 × 2 mm, FOV of 220, repetition time 6470 ms; echo time 105 ms) were aligned with the hippocampus and positioned perpendicular to the hippocampal coronal sequences with differing phase-encoding directions to evaluate the medial temporal lobes with reduced artifacts. Maps of the apparent diffusion coefficient (ADC) were obtained by a linear least-squares fit on a pixel-by-pixel basis after averaging of the direction-dependent DW images (Additional file 1). ADC maps and DW, T2-weighted images were analyzed for acute and chronic abnormalities [45]. The study protocol was approved by the local ethical committee. Written informed consent was received from participants or their legal designees prior to inclusion in the study (Ethics Committee of the Medical Faculty of Heidelberg Trial-Code No. S-248/2013).

### Endotoxic challenge

All experimental procedures and protocols used in this study were reviewed and approved by the Governmental Animal Protection Committee (Protocol Code No. 35–9185 81/G-147/12). Adult Wistar rats, weighing 250–350 g were kept in a 12 h day/light cycle and had free access to food and water. Endotoxemia was induced by injecting animals with 6 mg/kg lipopolysaccharide (LPS, Sigma) i.p. and returning them to the cage. Control animals did not receive any treatment. Vital functions of the pretreated animals were visually monitored every hour. Six hours after LPS injection, rats were sacrificed. This technique provided stable and reproducible experimental conditions, improving our comparison between treatment groups. Alternative animal sepsis models such as peritonitis induced by bacterial inoculum or cecal ligation and perforation (CLP) are well established approaches, however, the ensuing immune reactions are more difficult to quantify, producing unnecessary experimental variability.

### Acute brain slice preparation

Brain slices were prepared either from LPS-pretreated or control rats. Rats were anesthetized with Sevoflurane (Abbott, Wiesbaden, Germany) by inhalation and killed by decapitation. The brain was rapidly removed and submerged in ice cold artificial cerebrospinal fluid (ACSF, in mM: NaCl, 125; KCl, 3.5; MgCl_2_, 1.3; NaH_2_PO_4_, 1.2; CaCl_2_, 2.4; glucose, 25; NaHCO_3_, 26; gassed with 95 O_2_ and 5 % CO_2_). A vibratome (CU65 Cooling Unit & HM650V Vibratome, Microm, Walldorf, Germany) was used to cut 300 μm thick horizontal hippocampal slices in ACSF maintained at 0 °C. Slices were collected and transferred to a holding chamber containing either ACSF or ACSF with physostigmine (10 μM, Dr. F. Köhler Chemie GmbH, Bensheim, Germany). Slices were maintained at 32 °C for the first 30 min and then returned to room temperature until used for recordings over the subsequent four hours.

### Patch-clamp recordings

Single slices were transferred to a recording chamber (PM-1, Warner Instruments, Hamden, CT, USA or PC-R, Siskiyou, OR, USA), secured with a platinum harp and completely submerged with continuously flowing (3 ml/min) ACSF. Whole-cell patch-clamp recordings were made from CA1 hippocampal pyramidal neurons. Patch electrodes (3–4 MΩ) were made from borosilicate glass (1.5 mm, WPI, Sarasota, FL, USA) and filled with a potassium gluconate based solution (containing in mM: K-gluconate, 105; KCl, 30; HEPES, 10; K_2_-phosphocreatine, 10; Mg_2_-ATP, 4; Na_3_-GTP, 0.3; 293 mOsm: pH 7.3 with KOH). Recordings were made with a Multiclamp 700A or 700B amplifier, digitized through a Digidata 1322A A/D converter and acquired using pClamp 9 software (Axon Instruments and Molecular Devices, CA, USA). Data analysis was performed using IGOR PRO software (Wavemetrics, Lake Oswego, OR). All membrane potentials have been corrected for the calculated junction potential of −11 mV (JPCalc program by Dr. Peter H. Barry).

### Stimulation protocols

Paired pulse recordings were performed in standard solutions using a stimulation intensity adjusted to produce an EPSC around 200 pA in amplitude. Whole-cell LTP recordings were made in voltage clamp mode with the holding membrane potential at −70 mV. Evoked excitatory post-synaptic currents (EPSCs) were recorded in response to 100 μs long constant current pulse stimuli from constant current bipolar stimulus isolator units (A365, World Precision Instruments, Sarasota, Florida, USA). Stimulus isolators were connected to an Ag/AgCl electrode in the recording chamber and a glass patch pipette filled with ACSF and placed onto the surface of the slice. Stimuli were evoked at 0.1 Hz at an intensity adjusted to produce a single EPSC with average amplitude of 100–200 pA. EPSCs in the paired and control pathways were stimulated 400 ms apart. One stimulating electrode was positioned in stratum radiatum, in a proximity of the patched cell. Second stimulating electrode was placed in stratum radiatum, 50–100 μm away from the patched cell. Stable baseline measurements were obtained for at least 10 min before LTP was induced. High frequency stimulation (HFS) protocols consisted of 100 Hz stimulations of 1 s (i.e., 100 stimuli), repeated 4 times at 60 s intervals. During the HFS cell was current-clamped at 0 pA. The average response amplitude 10 min before LTP induction was taken as the baseline, and all values were normalized to this number. All recordings were made at room temperature.

### Statistics

Normal distribution of the data was tested using D’Agostino and Pearson omnibus normality test. One-way ANOVA followed by Tukey’s or Sidak’s multiple comparisons tests were performed using GraphPad Prism version 6.0f for Mac (GraphPad Software, La Jolla California USA, www.graphpad.comworks). The statistical analysis has been corrected for repeated measures. The sample size for each experiment was determined by power calculations based on previous experience with similar experiments. Data are presented as mean ± SEM.

## Results

### Hippocampal changes observed in MRI scans of patients with septic delirium

**Fig. 1:**
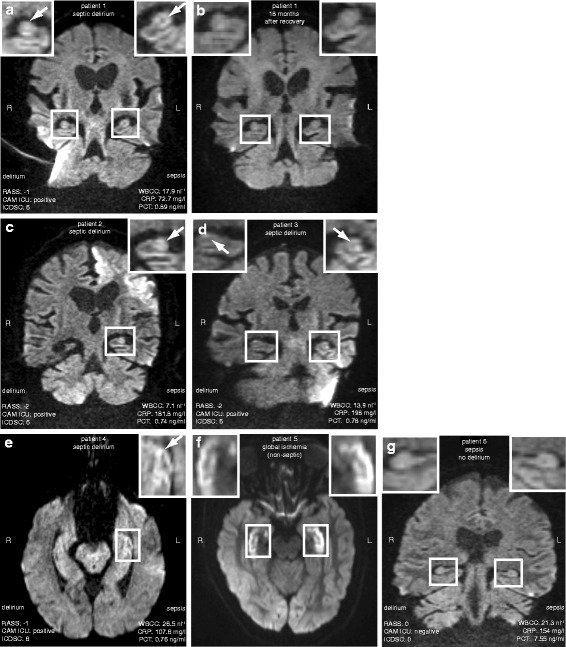
Pathologic signal changes in diffusion-weighted magnetic resonance imaging (DWI MRI) of the hippocampal formation in sepsis-induced delirium in human patients. **a**, **b**, **c**, **d**, **e** Images show coronal (**a**, **b**, patient 1 and **c**, patient 2, **d**, patient 3) and horizontal (**e**, patient 4) diffusion-weighted MRI scans of patients with sepsis and sepsis-induced delirium. Scores for delirium and sepsis shown in (panels **a**, **c**, **d**, **e** and **g**) were obtained within 24 h from the MRI scan. Boxed areas of the hippocampal formation are shown on an enlarged view in the top of each panel. White arrows indicate localized hyperintense signal elevations. **b** A scan taken from patient 1 eighteen months later shows no hyperintensities. The broad signal elevation in the left temporal lobe of patient 2 (**c**) was due to a previous occlusion of the left medial cerebral artery. **f** A scan from a non-septic patient with global cerebral hypoxia exhibits bilateral and broad signal elevation observed in the hippocampal formation. **g** Hippocampal hyperintensities were absent in septic patient without delirium. Hyperintense movement artifacts (right cerebellar area in a, left cerebellar area in **d**) or susceptibility artifacts (left temporal bone area in **f**) are occasionally observed in the DWI MRI scans. L: left; R: right; RASS: Richmond agitation sedation scale; CAM ICU: Confusion assessment method for intensive care unit; ICDSC: Intensive care delirium screening checklist; WBCC: white blood cell count; CRP: c-reactive protein; PCT: procalcitonin

Critically ill human patients, diagnosed with severe sepsis and septic encephalopathy (Additional file 2) received serial diffusion-weighted MRI (DWI MRI) brain scans. The results revealed localized hyperintense signals in the hippocampal formation (Fig. 1a, c, d, e). Restricted diffusion in the lateral hippocampus was observed in the DWI MRI of all four septic patients, independent of their disease history or secondary diagnoses, such as signal elevation in the left frontal lobe of one patient due to pre-existing ischemic stroke (Fig. 1c). DWI signal abnormality, therefore, could suggest a functional change in the hippocampus of patients diagnosed with septic delirium. A DWI MRI brain scan taken 18 months later in one patient, who had recovered from sepsis and septic encephalopathy, confirmed that the hippocampal hyperintensities were reversible (Fig. 1b). This does not exclude however, that other factors contributed to the observed hippocampal lesions. Patients with critical illness often suffer from impaired vascular perfusion. Hyperintense lesions in the hippocampus can also arise from global hypoxic/ischemic changes, a local vascular occlusion provoked by an embolism, or as a consequence of systemic hypoperfusion. Indeed, a DWI scan of a non-septic patient, suffering from global hypoxic damage revealed a more intense signal elevation affecting more of the hippocampus but still confined to this region (Fig. 1f). Thus septic delirium, like global hypoxia, causes the hippocampus to become hyperintense on DWI MRI scans. In septic patients without delirium such hippocampal hyperintensities were absent as shown in the DWI scan from a patient with sepsis but without delirium (Fig. 1g). These findings suggest that the hippocampal formation undergoes functional changes during global ischemia or sepsis with associated delirium. Thus hippocampal dysfunction may correlate with or be indicative of delirium among septic patients and may play an important role in the pathomechanism underlying septic delirium.

### Studying hippocampal cell function in an animal model of sepsis

**Fig. 2:**
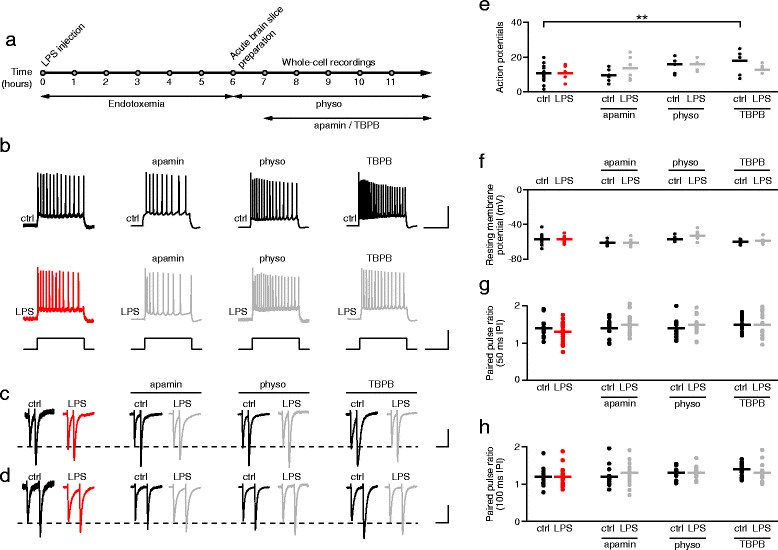
Basic properties of hippocampal CA1 pyramidal cells remain stable in lipopolysaccharide (LPS) treated rats. **a** Schematic diagram illustrates the experimental sequence used. LPS injection (6 mg/kg i.p.) occurred 6 h before slicing. Apamin (100 nM), physostigmine (10 μM, physo) and TBPB (3 μM) were applied to brain slices in vitro. **b** Representative current clamp recordings showing action potentials in response to a depolarizing current injection step (+300 pA, 1 s). Scale bars: 50 mV, 500 pA, 500 ms. **c**, **d** Representative EPSC responses to a paired-pulse stimulation protocol with 50 ms (**c**) and 100 ms (**d**) inter-pulse intervals (scale bars: 50 pA, 100 ms). **e**, **f** Summary data from all cells showing the number of APs during a 1 s 300 pA depolarizing current injection (**e**) and resting membrane potential **f**. ** *p* < 0.01, ANOVA followed by Dunnett’s multiple comparisons test. **g**, **h** Scatter plots of the paired pulse ratios (amplitude EPSC2/amplitude EPSC1) measured from all cells using 50 ms (**g**) and 100 ms (**h**) inter-pulse intervals. Bars represent mean values (**e**, **f**, **g**, **h**). Control: *n* = 15 cells; LPS: *n* = 10 cells; control + apamin: *n* = 6 cells; LPS + apamin: *n* = 8 cells; control + physo: *n* = 6 cells; LPS + physo: *n* = 5 cells; control + TBPB: *n* = 7 cells; LPS + TBPB: *n* = 7 cells. TBPB: [1-(1′-2-methylbenzyl)-1,4′-bipiperidin-4-yl)-1H-benzo[d]imidazol-2(3H)-one]

To further investigate the cellular mechanisms of sepsis-induced hippocampal dysfunction, we induced endotoxemia in rats, an animal model for sepsis, and performed single cell patch clamp electrophysiology experiments in the hippocampus of these rats. Wistar rats (250–350 g weight) received 6 mg/kg body weight lipopolysaccharide (LPS) i.p. and were returned to their cage to recover. After 6 h of endotoxemia, rats were sacrificed and acute brain slices were prepared for electrophysiological recordings (Fig. 2a). In addition to studying the effects of endotoxemia in LPS treated rats we included an analysis of the effects of enhancers of cholinergic activity since they are known to reduce cognitive deficits associated with both central anticholinergic syndrome and sepsis-induced delirium (see Introduction). Slices from control and LPS treated rats were thus treated in vitro for 2 h with either physostigmine (10 μM), a reversible cholinesterase inhibitor able to cross blood–brain barrier or TBPB [1-(1′-2-methylbenzyl)-1,4′-bipiperidin-4-yl)-1H-benzo[d]imidazol-2(3H)-one], a highly selective muscarinic M1 receptor allosteric agonist, or apamin, a blocker of SK2 channels which are known to be inhibited by muscarinic M1 subtype receptor activation in the rat hippocampus [13, 29]. Somatic whole cell patch clamp recordings were established from pyramidal neurons in the CA1 region of the hippocampus whose identity was confirmed from their characteristic action potential (AP) firing pattern in response to depolarizing current steps (Fig. 2b, Additional file 3). Except for more APs observed in the control rats after TBPB treatment, there were no differences in the number of APs induced by a 300 pA step between any of the treatment conditions (Fig. 2b, e, Additional file 3). The resting membrane potential of hippocampal neurons was also not significantly affected by LPS treatment or in vitro treatment with apamin, physostigmine or TBPB (Fig. 2f, Additional file 3).

To detect any changes in presynaptic function and neurotransmitter release probability we performed paired-pulse ratio analysis. Paired-pulse facilitation is a form of short-term plasticity, mainly of presynaptic origin, and very sensitive to presynaptic functional perturbations. Using a paired-pulse stimulation protocol with two different inter-pulse intervals (50 ms, Fig. 2c and 100 ms, Fig. 2d, Additional file 3), we found that the paired-pulse ratio did not significantly differ between any of the treatment groups (Fig. 2g, h, Additional file 3).

In summary, the analysis of resting membrane potential and paired-pulse ratios revealed no significant effects of LPS treatment, apamin, physostigmine or TBPB in vitro treatment on hippocampal pyramidal neurons. The analysis of cell excitability showed an elevated AP frequency in TBPB treated control cells.

### LPS treatment augments the apamin-sensitive component of the AHP of hippocampal CA1 neurons

The analysis of APs recorded from CA1 pyramidal neurons revealed altered afterhyperpolarization (AHP) in LPS treated rats. An AP in hippocampal pyramidal cells is followed by an AHP which can be divided into three components: a fast AHP (lasting 2–5 ms), medium AHP (lasting 50–100 ms) and a slow AHP (lasting longer than 1 s) [50]. Medium AHPs are mediated by SK channels and the apamin-sensitive SK2 subunit has been shown to be abundantly expressed in hippocampal CA1 pyramidal neurons [51].

**Fig. 3:**
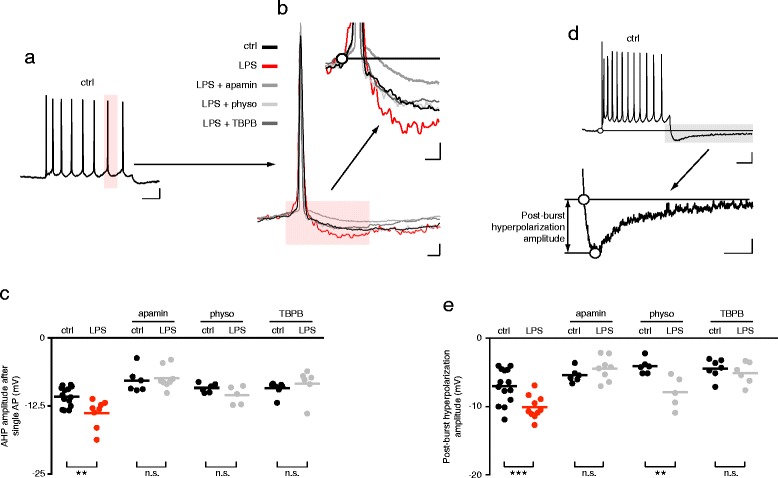
LPS treatment modulates action potential (AP) afterhyperpolarization (AHP) of the hippocampal neurons. **a** Example trace of AP firing upon depolarizing 300 pA current injection in a control rat (ctrl, and Fig. 2b). AHP analysis was performed on the second last AP (scale bars: 10 mV, 200 ms). **b** Representative APs from CA1 pyramidal cells of each treatment group, superimposed and aligned to the peak amplitude of the spike (lower panel, scale bars: 5 mV, 10 ms) and the spike firing threshold (white circle, upper panel, scale bars: 2 mV, 10 ms). **c**, **e** Scatter plots show peak amplitude from all cells. Bars are means. **d** The post-burst hyperpolarization amplitude was quantified relative to the pre-burst resting membrane potential and the post-burst hyperpolarizing peak voltage. (scale bars: upper panel 10 mV, 200 ms; lower panel 2 mV, 200 ms). n.s.: not significant, ** *p* < 0.01, *** *p* < 0.001, ANOVA followed by Sidak’s multiple comparisons test. Control: *n* = 14 cells; LPS: *n* = 8 cells; control + apamin: *n* = 6 cells; LPS + apamin: *n* = 8 cells; control + physo: *n* = 6 cells; LPS + physo: *n* = 5 cells; control + TBPB: *n* = 7 cells; LPS + TBPB: *n* = 6 cells

We examined the AHPs of the second last AP evoked by a 1 s 300 pA current injection (Figs. 2b and 3a). The peak amplitude of the AHP measured 2–100 ms after repolarization was increased in the LPS treated group, as compared to the control group (Fig. 3a-c, Additional file 3). The increased AHP amplitude in the LPS pretreated group peaked at a time point 50 ms after AP repolarization suggesting enhanced SK channel activation in the LPS treatment group. Indeed, acute treatment of these cells from LPS treated rats with bath applied apamin (100 nM), an SK2 channel antagonist, reduced AHP amplitude to below that of the control group. The apamin treatment of control rats revealed comparable results (Fig. 3c, Additional file 3).

The hyperpolarization observed following a series of APs (often termed an AP burst) is much larger than that following a single AP due to the removal of the 300 pA depolarizing current injection. We therefore also analyzed the post-burst hyperpolarization to assess any effects of LPS treatment (Fig. 3d, e, Additional file 3). Indeed, burst induced hyperpolarization was significantly increased in the LPS treated group. Application of apamin to the LPS pretreated group greatly reduced the amplitude of this hyperpolarization verifying that SK channels are strongly activated by a series of APs (Fig. 3e, Additional file 3).

In conclusion this data shows that the LPS treatment increased an AP afterhyperpolarization as well as hyperpolarization following an AP burst recorded from CA1 pyramidal neurons. Application of apamin reversed this effect, suggesting that enhanced activation of SK2 channels mediates the LPS treatment-induced increase of AHPs and post burst hyperpolarization.

### Apamin-sensitive AHP enhancement in LPS treated rats is reduced by boosting cholinergic activity

To assess the effect of anticholinesterases in treating sepsis induced delirium (see Introduction) we tested the effect of physostigmine in our rat sepsis model. We found that in vitro physostigmine application restores the LPS-induced enhancement of AHP peak amplitude (Fig. 3b, c, e, Additional file 3).

Since the muscarinic M1 subtype of cholinergic receptors inhibit apamin-sensitive SK2 channels in the rat hippocampus [13] we tested whether the muscarinic M1 receptor allosteric agonist TBPB could restore the effect of LPS treatment on AHPs. Indeed the enhanced AHP seen after LPS treatment was reversed by TBPB (Fig. 3b, c, e, Additional file 3).

Taken together, these results suggest that LPS exposure upregulates the apamin-sensitive SK2 channels, responsible for shaping medium AHPs and that this effect can be reversed by increasing endogenous acetylcholine activity or applying an M1 receptor agonist.

### Impaired hippocampal LTP in LPS treated rats is partly rescued by activation of M1 muscarinic acetylcholine receptors

**Fig. 4:**
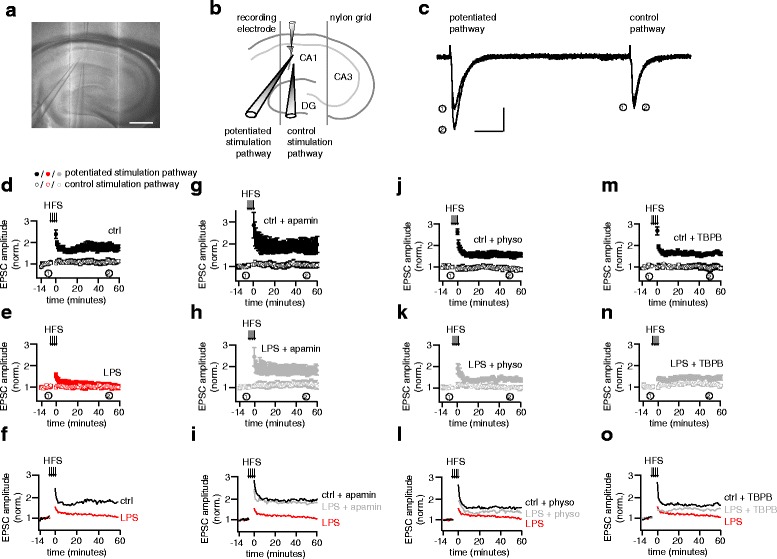
LPS treatment abolishes cellular LTP in the Schaffer collaterals, an effect partly rescued by activation of M1 muscarinic acetylcholine receptors. A 4× magnification image (**a**, scale bar 400 μm) and schematic representation (**b**) of an acute brain slice showing the recording (top) and stimulating (bottom left and middle) glass electrodes, DG: dentate gyrus. **c** Overlay of typical EPSC recordings evoked by stimulating the control and potentiation pathways during the baseline acquisition (1) and 50 min after LTP induction with high frequency stimulation (HFS: 4 × 100 pulses at 100 Hz every 60 s, scale bars: 100 pA, 50 ms) (2). **d**, **e**, **g**, **h**, **j**, **k**, **m**, **n** Time series plots show the mean (± SEM) EPSC amplitudes normalized to baseline for all cellular LTP experiments. (**f**, **i**, **l**, **o**) Overlaid timeplots of the mean EPSC amplitude data shown in (**d**, **e**, **g**, **h**, **j**, **k**, **m**, **n**). Control: *n* = 15 cells; LPS: *n* = 10 cells; control + apamin: *n* = 6 cells; LPS + apamin: *n* = 8 cells; control + physo: *n* = 6 cells; LPS + physo: *n* = 8 cells; control + TBPB: *n* = 7 cells; LPS + TBPB: *n* = 7 cells

Given the observed alteration in hippocampal function in septic patients (Fig. 1), we postulated that the systemic inflammation, induced by LPS injection, would affect hippocampal synaptic plasticity, a phenomenon, reported to be critical for learning and memory [34]. To this end we recorded from CA1 neurons and measured excitatory postsynaptic currents (EPSCs) in response to two stimulating glass pipettes placed on the Schaffer collateral pathway from the CA3 region (Fig. 4a, b, c). Synaptic inputs stimulated by one pipette were potentiated with 4 bursts of high frequency stimulation while the second pipette served as a control pathway to monitor the stability of the recording. Indeed, cellular LTP, which persisted for more than 60 min after its induction in slices from control rats was abolished in slices obtained from rats pretreated with LPS (Fig. 4d-f, Additional file 3). In vitro application of apamin (100 nM) to the LPS pretreated rats resulted in a full rescue of LTP (Fig. 4g-i, Additional file 3). Since physostigmine and TBPB reversed the effects of LPS treatment on the SK channel mediated AHP, we next asked whether they could similarly rescue the LPS-induced deficit in LTP. Application of 5 μM and 10 μM physostigmine into the bath solution immediately prior to the recordings did not rescue the LPS induced deficit in LTP (Additional file 4). However, addition of physostigmine (10 μM) to the slice holding chamber at least 2 h before the recording and during the recording partly rescued the LTP deficit in LPS treated rats (Fig. 4j-l, Additional file 3). Application of TBPB to the slices immediately prior to the recordings also partly rescued the loss of LTP observed in the LPS treated rats (Fig. 4m-o, Additional file 3).

**Fig. 5:**
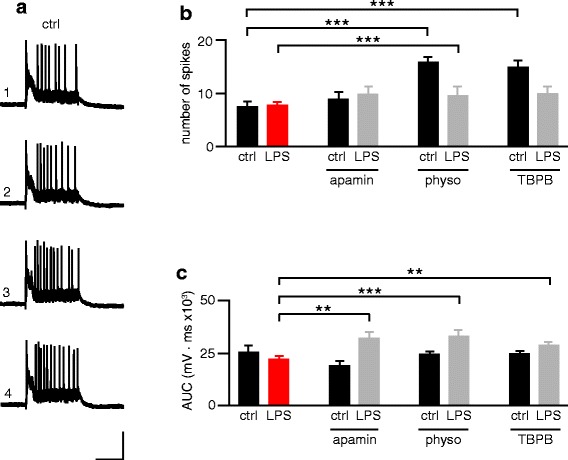
LPS treatment does not affect spike numbers or depolarization induced with high frequency stimulation. **a** Representative traces showing AP bursts induced by 4 successive high frequency stimulations (HFS, 100 Hz for 1 s) in the control rat (ctrl, scale bars: 50 mV, 500 ms. Histograms show the mean ± SEM values of the total number of spikes (**b**) and total AUC (**c**) from all 4 HFS trains used to induce LTP in Fig. 4. ** *p* < 0.01, *** *p* < 0.001 (ANOVA followed by Dunnett’s multiple comparisons test). Control: *n* = 8 cells; LPS: *n* = 10 cells; control + apamin: *n* = 6 cells; LPS + apamin: *n* = 8 cells; control + physo: *n* = 6 cells; LPS + physo: *n* = 6 cells; control + TBPB: *n* = 7 cells; LPS + TBPB: *n* = 7 cells

To further investigate the mechanism by which SK channels appear to mediate reduced LTP in LPS treated rats, we analyzed the AP bursts induced by the high frequency stimulation protocol used to induce LTP. These postsynaptic responses consist of a burst of APs superimposed on a prolonged baseline depolarization (Fig. 5a). Analysis of the number of spikes and the AUC during the entire AP burst did not reveal any effect of LPS treatment on the postsynaptic response to LTP-inducing stimulation (Fig. 5b, c, Additional file 5). An increased AP number was observed in the control groups treated with physostigmine (Fig. 5b, Additional file 5) and TBPB (Fig. 5b, Additional file 5, Additional file 3). From the AUC analysis, an increase specifically in the LPS treated rats was seen after apamin, physostigmine and TBPB treatment. This data suggests that apamin, physostigmine and TBPB increase the postsynaptic response to synaptic stimulation in terms of either action potential generation or depolarization or both. LPS treatment alone, however, did not affect AP generation or depolarization of these synaptically-induced, LTP-generating bursts.

Taken together, our data show an endotoxin induced disruption of synaptic plasticity in the rat brain accompanied by an increased SK channel-mediated AHP. Inhibiting SK channel function with either the specific blocker, apamin, or with an M1 muscarinic acetylcholine receptor activation or by increasing the lifetime of endogenous acetylcholine with cholinesterase inhibitors can partly restore the deficit in synaptic plasticity induced by sepsis.

## Discussion

In this study we observed functional changes in the hippocampus of patients diagnosed with sepsis-associated encephalopathy by using DWI MRI. In an animal model for sepsis, LPS-induced endotoxemia, we also show hippocampal dysfunction in the form of a deficit in synaptic plasticity as well as an increase in a component of the AHP presumably mediated by SK channels. The partial rescue of the effects of endotoxemia by increasing endogenous cholinergic activity or applying an exogenous M1 receptor agonist in our animal model identify pharmacological targets for treatment of sepsis induced delirium in patients in the ICU. Potential mechanisms causing increased SK activity following endotoxemia include reduced central cholinergic function, increased SK channel expression or increased calcium influx following synaptic activity.

Previous studies have shown that the septic brain undergoes functional and structural changes [5]. However, a region specific approach, in particular hippocampal function has not been thoroughly described. The implementation of a DWI scan protocol, commonly used in the field of epilepsy and stroke research, allows for improved spatial and functional analysis of the hippocampus [53] and may prove useful to identify sepsis associated encephalopathy in septic delirium patients in the ICU. However, only a combined approach, including laboratory tests, clinical examination, clinical scores as well as diagnostic imaging can verify a clinical diagnosis of this disorder.

Our DWI MRI analysis has identified the hippocampus as the site of dysfunction and pathology in sepsis-induced delirium. Cognitive deficits often associated with delirium during sepsis include spatial and temporal disorientation, confusion as well as impaired learning and memory. These clinical features are typically associated with hippocampal dysfunction. Nonetheless, conditions such as hypoxia, hypercarbia, hypotension or pharmacotherapy with drugs affecting brain function should be taken in account when interpreting cognitive disorders in septic patients.

Sepsis-induced delirium and global ischemia both reduce hippocampal ADC. This hippocampus specific pathology of both sepsis and global cerebral ischemia suggests that hypoperfusion may also play a role in the pathomechanism of sepsis-induced delirium. Indeed imbalances in the sympathetic and parasympathetic nervous system in sepsis and septic shock lead to hypotension which can result in organ hypoperfusion and ischemia. Thus brain hypoperfusion and any resulting brain ischemia could contribute to the cognitive deficits associated with hippocampal dysfunction in sepsis induced delirium.

Endotoxin, administered peripherally in rats did not affect hippocampal neuron resting membrane potential, firing patterns or short term synaptic plasticity but augmented SK channel function and AHP amplitude. Although basal excitability was unaffected in this septic state, the calcium influx, caused by a burst of synaptic activity, more strongly activated SK channels to increase post-burst hyperpolarization which most likely caused the reduction in long term synaptic potentiation, the cellular mechanism widely believed to underlie memory. In line with this, the LPS-induced increase in AHP was reversed, and the deficit in synaptic plasticity was partly restored by in vitro pharmacological enhancement of cholinergic neurotransmission, which is known to inhibit SK function via M1 receptor activation. Cholinergic conditioning of an animal, during the initial 6 h after LPS injection, might more effectively rescue the LTP deficits through both central and peripheral mechanisms. Vagal stimulation has been shown to restore synaptic function and reduce cytokine production in endotoxemia [11, 24] and our group has previously shown that systemic physostigmine reduces the capillary leakage and the leukocyte-endothelial interaction caused by endotoxemia [41]. A cholinergic deficiency hypothesis involving reduced tone in the autonomic nervous system has emerged to explain such results. A central deficit in cholinergic activity has also been implicated by results showing increased anticholinesterase activity in the brain during endotoxemia [54]. Our current result showing a partial rescue of the LPS-induced deficit in LTP in rat brain slices identifies a central anti-inflammatory effect of anticholinesterase inhibitors and is consistent with a sepsis-induced cholinergic deficiency in the central nervous system which parallels the reduced vagal tone in the periphery. However, causal evidence for a central cholinergic deficiency in sepsis is lacking.

Diverse mechanisms of cholinergic modulation of synaptic plasticity have been proposed both for in vitro and in vivo experimental settings [2, 12, 15, 55]. In particular, the M1 subtype of muscarinic acetylcholine receptors has been shown to play important role in shaping the plastic changes of hippocampal excitatory synapses [48]. The ability of muscarinic M1 receptor subunits to indirectly affect LTP by inhibiting SK2 channels has been proposed as a mechanism underlying cholinergic control of synaptic plasticity [8, 13, 25]. In fact, we were able to demonstrate that this mechanism might play a crucial role in the septic brain. Indeed, the SK blocker apamin has been shown to be of protective benefit in the treatment of septic shock in mice when injected prior to LPS [14]. Both TBPB and physostigmine caused increased number of action potentials evoked by high frequency stimulation, reduced AHP and post burst hyperpolarisation without affecting resting membrane potential or paired pulse ratio. These effects of TBPB and physostigmine indicate increased excitability due to cholinergic M1 receptor-induced suppression of SK independent of the presence of sepsis. A more input specific or sepsis-selective cholinergic boost may be necessary to avoid side-effects and more fully restore endotoxemia-induced deficits in LTP. Such side-effects and a lack of selectivity may be related to the failure of rivastigmine in recent human trials [19].

It is unlikely that the deficits in LTP which we show are an anomaly of our endotoxemia model of sepsis. A deficit in Schaffer collateral LTP has also been shown to occur following septic encephalopathy induced by cecal ligation and puncture (CLP) in mice [28]. Thus a diminished capacity for hippocampal synaptic potentiation appears to be a consequence of sepsis independent of the animal sepsis model used.

The N-Methyl-D-aspartate (NMDA) subtype of glutamate receptors play a crucial role in the induction of cellular LTP by allowing Ca^2+^ influx into the postsynaptic dendritic spines during LTP induction. This leads to the activation of intracellular cascades such as calcium/calmodulin-dependent protein kinase II (CaMKII), resulting in increased postsynaptic responses [16]. Besides the ability to shape AHPs, SK2 channels are known to interact with postsynaptic NMDA receptors in an activity-dependent feedback manner resulting in the rapid Mg^2+^ block of the NMDA channels [1, 35]. Thus the mechanism by which SK2 channel blockade promotes LTP or rescues LPS-induced deficits in LTP may be via promoting NMDA receptor activation during AP bursts.

## Conclusions

To conclude, our findings using an animal model of sepsis, point to a dysfunction in a calcium-activated potassium channel in the hippocampus which most likely underlies plasticity deficits in rats and could be involved in sepsis-induced delirium in humans. Furthermore, we propose that increased activation of cholinergic M1 receptors, which rescued LTP deficits in our rat model, might be beneficial in the therapeutic treatment of septic delirium in the ICU.

### Ethical approval

All procedures performed in studies involving human participants were in accordance with the ethical standards of the institutional and/or national research committee and with the 1964 Helsinki declaration and its later amendments or comparable ethical standards. All applicable international, national, and/or institutional guidelines for the care and use of animals were followed. All procedures performed in studies involving animals were in accordance with the ethical standards of the institution or practice at which the studies were conducted.

## Additional files

Additional file 1:
**ADC maps corresponding to the diffusion-weighted magnetic resonance images (DWI MRI) of the hippocampal formation from patients 1, 2, 3, 4 and 6 (a-e) shown in Fig.** **1** **.** (PDF 2079 kb) Additional file 2:
**Patient medical condition prior to MRI scan.** Vital parameters, list of relevant medication and the laboratory results of the patient are recorded before receiving MRI diagnostics. (PDF 81 kb) Additional file 3:
**Results summary.** Tables show measured values from all experiments in this study. Data is presented as mean ± SEM. (PDF 31 kb) Additional file 4:
**Physostigmine without preincubation fails to rescue LTP induced by LPS treatment.** Time series plots as described in the Fig. 4 but with physostigmine (a: 5 μM, *n* = 3 cells; b: 10 μM *n* = 2 cells) applied only during the recordings without preincubation. (PDF 265 kb) Additional file 5:
**LPS treatment does not affect spike numbers or depolarization induced with high frequency stimulation. Scatter plots represent number of spikes (a, b, e, f, i, j, m, n) and area under curve (AUC, c, d, g, h, k, l, o, p) per HFS recorded from all cells.** Bars are mean values. Control: *n* = 8 cells; LPS: *n* = 10 cells; control + apamin: *n* = 6 cells; LPS + apamin: *n* = 8 cells; control + physo: *n* = 6 cells; LPS + physo: *n* = 6 cells; control + TBPB: *n* = 7 cells; LPS + TBPB: *n* = 7 cells. (PDF 354 kb)
