# Supplementary figures and images for: Muscarinic M1 receptors modulate endotoxemia-induced loss of synaptic plasticity
Source: Acta Neuropathol Commun. 2015 Nov 4;3:67. doi: 10.1186/s40478-015-0245-8 (PMC4632469; doi:10.1186/s40478-015-0245-8)

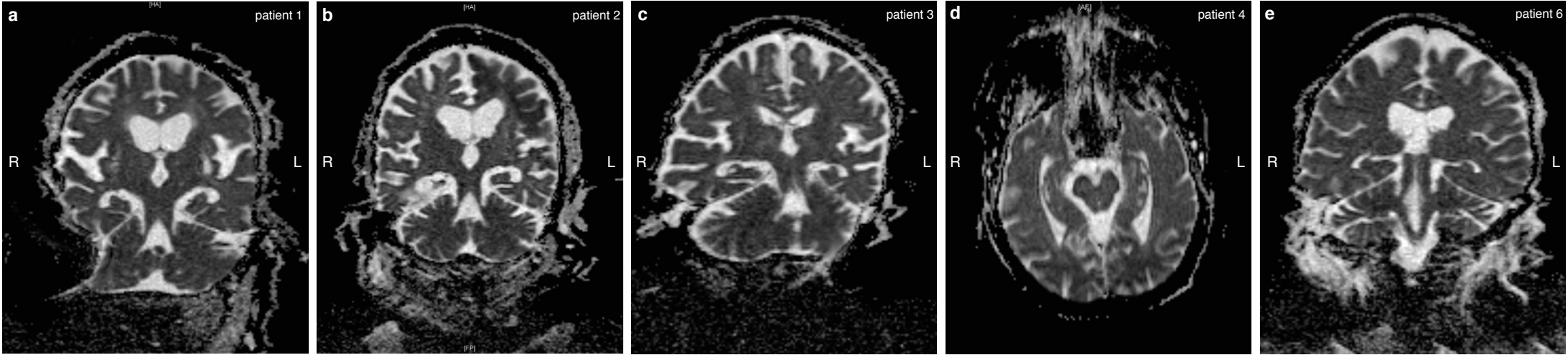

Supplement: Additional file 1: — ADC maps corresponding to the diffusion-weighted magnetic resonance images (DWI MRI) of the hippocampal formation from patients 1, 2, 3, 4 and 6 (a-e) shown in Fig. 1 . (PDF 2079 kb) [file 40478_2015_245_MOESM1_ESM.pdf]

Additional file 4

a

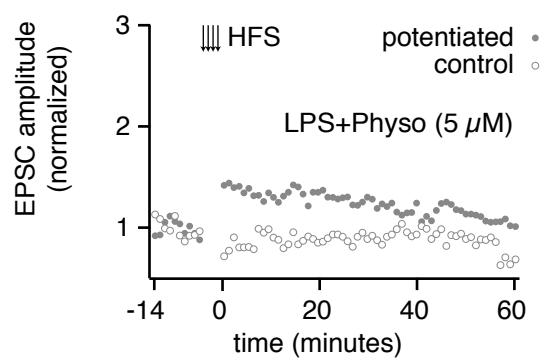

b

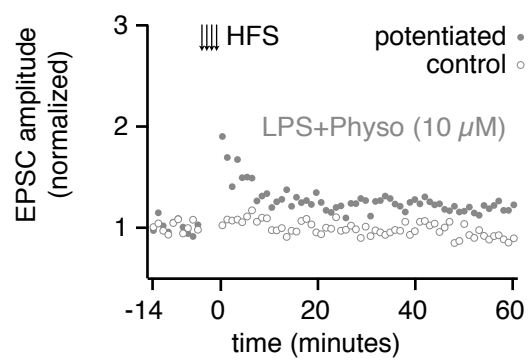

Supplement: Additional file 4: — Physostigmine without preincubation fails to rescue LTP induced by LPS treatment. Time series plots as described in the Fig. 4 but with physostigmine (a: 5 μM, n = 3 cells; b: 10 μM n = 2 cells) applied only during the recordings without preincubation. (PDF 265 kb) [file 40478_2015_245_MOESM4_ESM.pdf]

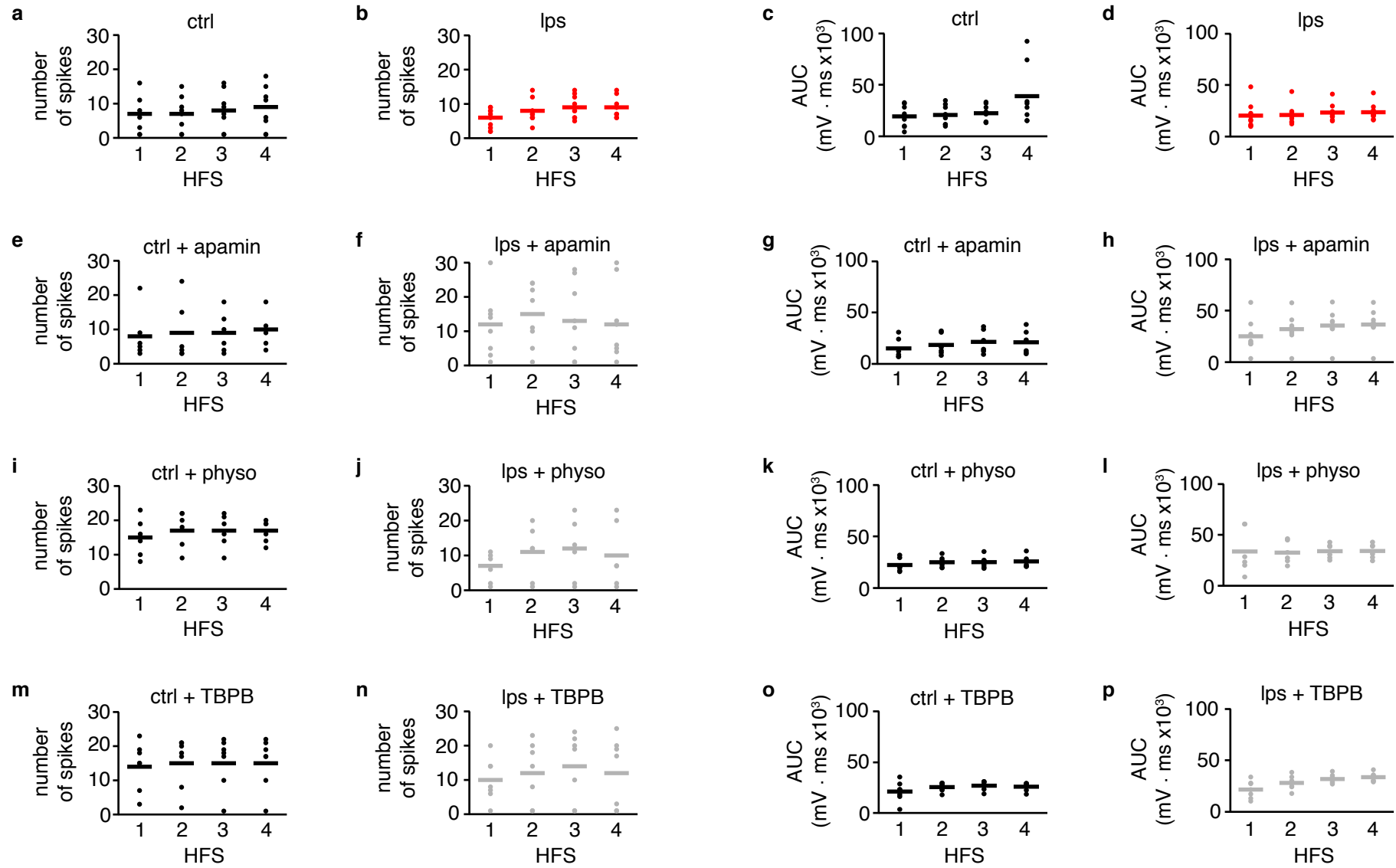

Supplement: Additional file 5: — LPS treatment does not affect spike numbers or depolarization induced with high frequency stimulation. Scatter plots represent number of spikes (a, b, e, f, i, j, m, n) and area under curve (AUC, c, d, g, h, k, l, o, p) per HFS recorded from all cells. Bars are mean values. Control: n = 8 cells; LPS: n = 10 cells; control + apamin: n = 6 cells; LPS + apamin: n = 8 cells; control + physo: n = 6 cells; LPS + physo: n = 6 cells; control + TBPB: n = 7 cells; LPS + TBPB: n = 7 cells. (PDF 354 kb) [file 40478_2015_245_MOESM5_ESM.pdf]
