# Supplementary material for: Muscarinic M1 receptors modulate endotoxemia-induced loss of synaptic plasticity
Source: Acta Neuropathol Commun. 2015 Nov 4;3:67. doi: 10.1186/s40478-015-0245-8 (PMC4632469; doi:10.1186/s40478-015-0245-8)
Supplement: Additional file 2: — Patient medical condition prior to MRI scan. Vital parameters, list of relevant medication and the laboratory results of the patient are recorded before receiving MRI diagnostics. (PDF 81 kb) [file 40478_2015_245_MOESM2_ESM.pdf]

Additional file 2

Patient #1

vital parameters

measurement	units	result
**haemodynamics**		
systolic arterial pressure	mmHg	131
diastolic arterial pressure	mmHg	68
mean arterial pressure	mmHg	88
central venous pressure	cmH_2O	6
heart rate	min^{-1}	67
**catecholamine**		
noradrenaline	µg/kg/min	0
vasopressin	U/kg/min	0
dobutamine	mg/kg/min	0
adrenaline	µg/kg/min	0
**other medication**		
hydrocortisone	mg/24h	0
furosemide	mg/24h	0
**sedation**		
dexmedetomidine	µg/kg/min	0.3
**fluid replacement**		
cristalloids	ml/24h	3429
colloids	ml/24h	0
enteral feeding	ml/24h	1410
red cell concentrate	ml/24h	0
fresh frozen plasma	ml/24h	0
thrombocyte concentrate	ml/24h	0
coagulation factors	U/24h	0
**fluid loss**		
urine	ml/24h	560
hemodialysis	ml/24h	4830
abdominal drainage	ml/24h	30
pleural drainage	ml/24h	0
**fluid balance**		
total	(+/-) ml/24h	-581
**scores**		
RASS		-1
CAM ICU		positive
ICDSC		6
**ventilation**		
fraction of inspired oxygen (FiO_2)		0.4
respiratory rate	min^{-1}	20
mode		CPAP

laboratory diagnostics

measurement	units	result
**infectiology**		
white blood cells	nl^{-1}	17.92
C-reactive protein (CRP)	mg/l	72.7
procalcitonin (PCT)	ng/ml	0.89
body temperature	°C	36.1
**clinical chemistry**		
red blood cells	pl^{-1}	2.8
haemoglobin	g/dl	8.4
haematocrit	%	0.25
thrombocytes	nl^{-1}	153
Quick	%	97.5
activated partial thromboplastin time (APTT)	s	29.3
creatinine	mg/dl	1.06
urea	mg/dl	44
blood glucose	mg/dl	145
triglyceride	mg/dl	/
cholesterol	mg/dl	/
aspartate transaminase (AST)	U/l	28
alanine transaminase (ALT)	U/l	42
gamma-glutamyltransferase (GGT)	U/l	162
cholinesterase (CHE)	kU/l	4.15
alkaline phosphatase (AP)	U/l	69
lactate dehydrogenase (LDH)	U/l	377
bilirubin	mg/dl	1.2
amylase	U/l	19
lipase	U/l	19
proteins	g/l	46.4
albumin	g/dl	28.7
sodium	mmol/l	133
potassium	mmol/l	4.9
calcium	mmol/l	2.21
magnesium	mmol/l	/
chloride	mmol/l	102
phosphate	mmol/l	/
**blood gas analysis**		
pH		7.36

vital parameters		
**measurement**	**units**	**result**
paO_2/FiO_2		417

laboratory diagnostics		
**measurement**	**units**	**result**
partial pressure of CO_2 in arterial blood ($paCO_2$)	mmHg	49
partial pressure of oxygen in arterial blood (paO_2)	mmHg	88
arterial oxygen saturation (SaO_2)	%	96
bicarbonate	mmol/l	24.9
base excess	mmol/l	0.7

Patient #2

vital parameters		
**measurement**	**units**	**result**
**haemodynamics**		
systolic arterial pressure	mmHg	118
diastolic arterial pressure	mmHg	55
mean arterial pressure	mmHg	77
central venous pressure	cmH_2O	24
heart rate	min^{-1}	63
**catecholamine**		
noradrenaline	µg/kg/min	0.054
vasopressin	U/kg/min	0
dobutamine	mg/kg/min	0
adrenaline	µg/kg/min	0
**other medication**		
hydrocortisone	mg/24h	0
furosemide	mg/24h	100
**sedation**		
clonidine	mg/h	0.06
**fluid replacement**		
cristalloids	ml/24h	4191.9
colloids	ml/24h	0
enteral feeding	ml/24h	90
red cell concentrate	ml/24h	0
fresh frozen plasma	ml/24h	0
thrombocyte concentrate	ml/24h	0
coagulation factors	U/24h	0
**fluid loss**		
urine	ml/24h	3165
hemodialysis	ml/24h	0
abdominal drainage	ml/24h	770
pleural drainage	ml/24h	0
**fluid balance**		
total	(+/-) ml/24h	+346.9
**scores**		
RASS		-2
CAM ICU		positive

laboratory diagnostics		
**measurement**	**units**	**result**
**infectiology**		
white blood cells	nl^{-1}	7.1
C-reactive protein (CRP)	mg/l	181.8
procalcitonin (PCT)	ng/ml	0.74
body temperature	°C	36.7
**clinical chemistry**		
red blood cells	pl^{-1}	2.7
haemoglobin	g/dl	8.1
haematocrit	%	0.25
thrombocytes	nl^{-1}	302
Quick	%	95.5
activated partial thromboplastin time (APTT)	s	35.8
creatinine	mg/dl	0.84
urea	mg/dl	94
blood glucose	mg/dl	103
triglyceride	mg/dl	172
cholesterol	mg/dl	/
aspartate transaminase (AST)	U/l	37
alanine transaminase (ALT)	U/l	36
gamma-glutamyltransferase (GGT)	U/l	496
cholinesterase (CHE)	kU/l	1.83
alkaline phosphatase (AP)	U/l	333
lactate dehydrogenase (LDH)	U/l	253
bilirubin	mg/dl	0.6
amylase	U/l	38
lipase	U/l	31
proteins	g/l	56.4
albumin	g/dl	26.2
sodium	mmol/l	134
potassium	mmol/l	4.36
calcium	mmol/l	1.97

vital parameters		
**measurement**	**units**	**result**
ICDSC		6
**ventilation**		
fraction of inspired oxygen (FiO_2)		0.3
respiratory rate	min^{-1}	14
mode		CPAP
paO_2/FiO_2		396.7

laboratory diagnostics		
**measurement**	**units**	**result**
magnesium	mmol/l	/
chloride	mmol/l	/
phosphate	mmol/l	/
**blood gas analysis**		
pH		7.36
partial pressure of CO_2 in arterial blood ($paCO_2$)	mmHg	39
partial pressure of oxygen in arterial blood (paO_2)	mmHg	119
arterial oxygen saturation (SaO_2)	%	99
bicarbonate	mmol/l	21.7
base excess	mmol/l	-3.3

Patient #3

vital parameters		
**measurement**	**units**	**result**
**haemodynamics**		
systolic arterial pressure	mmHg	139
diastolic arterial pressure	mmHg	63
mean arterial pressure	mmHg	93
central venous pressure	cmH_2O	15
heart rate	min^{-1}	97
**catecholamine**		
noradrenaline	µg/kg/min	0.032
vasopressin	U/kg/min	0
dobutamine	mg/kg/min	0
adrenaline	µg/kg/min	0
**other medication**		
hydrocortisone	mg/24h	0
furosemide	mg/24h	80
**sedation**		
		none
**fluid replacement**		
cristalloids	ml/24h	3700
colloids	ml/24h	0
enteral feeding	ml/24h	420
red cell concentrate	ml/24h	0
fresh frozen plasma	ml/24h	0
thrombocyte concentrate	ml/24h	0
coagulation factors	U/24h	0
**fluid loss**		
urine	ml/24h	2200
hemodialysis	ml/24h	0
abdominal drainage	ml/24h	120

laboratory diagnostics		
**measurement**	**units**	**result**
**infectiology**		
white blood cells	nl^{-1}	13.92
C-reactive protein (CRP)	mg/l	198
procalcitonin (PCT)	ng/ml	0.78
body temperature	°C	37.4
**clinical chemistry**		
red blood cells	pl^{-1}	2.7
haemoglobin	g/dl	8.1
haematocrit	%	0.25
thrombocytes	nl^{-1}	209
Quick	%	73.5
activated partial thromboplastin time (APTT)	s	40.6
creatinine	mg/dl	1.57
urea	mg/dl	127
blood glucose	mg/dl	105
aspartate transaminase (AST)	U/l	28
alanine transaminase (ALT)	U/l	22
gamma-glutamyltransferase (GGT)	U/l	394
alkaline phosphatase (AP)	U/l	239
lactate dehydrogenase (LDH)	U/l	239
bilirubin	mg/dl	1.0
amylase	U/l	13
lipase	U/l	26
albumin	g/dl	18
sodium	mmol/l	142

vital parameters		
measurement	units	result
pleural drainage	ml/24h	0
**fluid balance**		
total	(+/-) ml/24h	+1800
**scores**		
RASS		-2
CAM ICU		positive
ICDSC		6
**ventilation**		
fraction of inspired oxygen (FiO_2)		0.45
respiratory rate	min^{-1}	16
mode		CPAP
paO_2/FiO_2		280

laboratory diagnostics		
measurement	units	result
potassium	mmol/l	4.74
calcium	mmol/l	1.48
chloride	mmol/l	112
**blood gas analysis**		
pH		7.42
partial pressure of CO_2 in arterial blood ($paCO_2$)	mmHg	39
partial pressure of oxygen in arterial blood (paO_2)	mmHg	107
arterial oxygen saturation (SaO_2)	%	96
bicarbonate	mmol/l	24.6
base excess	mmol/l	0.1

Patient #4

vital parameters		
measurement	units	result
**haemodynamics**		
systolic arterial pressure	mmHg	130
diastolic arterial pressure	mmHg	50
mean arterial pressure	mmHg	75
central venous pressure	cmH_2O	5
heart rate	min^{-1}	82
**catecholamine**		
noradrenaline	μg/kg/min	0
vasopressin	U/kg/min	0
dobutamine	mg/kg/min	0
adrenaline	μg/kg/min	0
**other medication**		
hydrocortisone	mg/24h	96
furosemide	mg/24h	0
**sedation**		
		none
**fluid replacement**		
cristalloids	ml/24h	1700
colloids	ml/24h	0
enteral feeding	ml/24h	270
red cell concentrate	ml/24h	0
fresh frozen plasma	ml/24h	0
thrombocyte concentrate	ml/24h	0
coagulation factors	U/24h	0
**fluid loss**		
urine	ml/24h	1060
hemodialysis	ml/24h	0
abdominal drainage	ml/24h	0

laboratory diagnostics		
measurement	units	result
**infectiology**		
white blood cells	nl^{-1}	26.52
C-reactive protein (CRP)	mg/l	107.6
procalcitonin (PCT)	ng/ml	0.76
body temperature	°C	37.6
**clinical chemistry**		
red blood cells	pl^{-1}	2.6
haemoglobin	g/dl	7.8
haematocrit	%	0.25
thrombocytes	nl^{-1}	263
Quick	%	63.5
activated partial thromboplastin time (APTT)	s	56
creatinine	mg/dl	1.04
urea	mg/dl	108
blood glucose	mg/dl	198
triglyceride	mg/dl	218
cholesterol	mg/dl	67
aspartate transaminase (AST)	U/l	47
alanine transaminase (ALT)	U/l	44
gamma-glutamyltransferase (GGT)	U/l	219
cholinesterase (CHE)	kU/l	1.7
alkaline phosphatase (AP)	U/l	122
lactate dehydrogenase (LDH)	U/l	446
bilirubin	mg/dl	1.2
amylase	U/l	13

vital parameters		
**measurement**	**units**	**result**
pleural drainage	ml/24h	220
**fluid balance**		
total	(+/-) ml/24h	-700
**scores**		
RASS		-1
CAM ICU		positive
ICDSC		6
**ventilation**		
fraction of inspired oxygen (FiO_2)		0.3
respiratory rate	min^{-1}	18
mode		CPAP
paO_2/FiO_2		333.3

laboratory diagnostics		
**measurement**	**units**	**result**
lipase	U/l	13
proteins	g/l	35
albumin	g/dl	18
sodium	mmol/l	150
potassium	mmol/l	4.8
calcium	mmol/l	1.88
magnesium	mmol/l	1.18
chloride	mmol/l	119
phosphate	mmol/l	1.22
**blood gas analysis**		
pH		7.39
partial pressure of CO_2 in arterial blood ($paCO_2$)	mmHg	43.5
partial pressure of oxygen in arterial blood (paO_2)	mmHg	100
arterial oxygen saturation (SaO_2)	%	97
bicarbonate	mmol/l	25.2
base excess	mmol/l	0.8

Patient #6

vital parameters		
**measurement**	**units**	**result**
**haemodynamics**		
systolic arterial pressure	mmHg	144
diastolic arterial pressure	mmHg	67
mean arterial pressure	mmHg	89
central venous pressure	cmH_2O	15
heart rate	min^{-1}	93
**catecholamine**		
noradrenaline	μg/kg/min	0.143
vasopressin	U/kg/min	0
dobutamine	mg/kg/min	0
adrenaline	μg/kg/min	0
**other medication**		
hydrocortisone	mg/24h	0
furosemide	mg/24h	0
**sedation**		
morphine	mg/h	4
clonidine	mg/h	0.06
**fluid replacement**		
cristalloids	ml/24h	4600
colloids	ml/24h	0
enteral feeding	ml/24h	1000
red cell concentrate	ml/24h	550

laboratory diagnostics		
**measurement**	**units**	**result**
**infectiology**		
white blood cells	nl^{-1}	21.25
C-reactive protein (CRP)	mg/l	154.2
procalcitonin (PCT)	ng/ml	7.55
body temperature	°C	36.2
**clinical chemistry**		
red blood cells	pl^{-1}	2.3
haemoglobin	g/dl	7.0
haematocrit	%	0.2
thrombocytes	nl^{-1}	70
Quick	%	60.6
activated partial thromboplastin time (APTT)	s	36.6
creatinine	mg/dl	0.85
urea	mg/dl	56
blood glucose	mg/dl	124
triglyceride	mg/dl	107
cholesterol	mg/dl	62
aspartate transaminase (AST)	U/l	59
alanine transaminase (ALT)	U/l	40

vital parameters		
**measurement**	**units**	**result**
fresh frozen plasma	ml/24h	0
thrombocyte concentrate	ml/24h	0
coagulation factors	U/24h	0
**fluid loss**		
urine	ml/24h	1300
hemodialysis	ml/24h	1300
abdominal drainage	ml/24h	760
pleural drainage	ml/24h	0
**fluid balance**		
total	(+/-) ml/24h	+2790
**scores**		
RASS		0
CAM ICU		negative
ICDSC		0
**ventilation**		
fraction of inspired oxygen (FiO_2)		0.65
respiratory rate	min^{-1}	15
mode		BIPAP
paO_2/FiO_2		101.5

laboratory diagnostics		
**measurement**	**units**	**result**
gamma-glutamyltransferase (GGT)	U/l	68
cholinesterase (CHE)	kU/l	0.61
alkaline phosphatase (AP)	U/l	216
lactate dehydrogenase (LDH)	U/l	611
bilirubin	mg/dl	0.6
amylase	U/l	<5
lipase	U/l	17
proteins	g/l	39.4
albumin	g/dl	22.9
sodium	mmol/l	144
potassium	mmol/l	4.6
calcium	mmol/l	2.02
magnesium	mmol/l	0.78
chloride	mmol/l	113
phosphate	mmol/l	1.01
**blood gas analysis**		
pH		7.42
partial pressure of CO_2 in arterial blood ($paCO_2$)	mmHg	41
partial pressure of oxygen in arterial blood (paO_2)	mmHg	62
arterial oxygen saturation (SaO_2)	%	98
bicarbonate	mmol/l	26.1
base excess	mmol/l	1.9
