# Supplementary material for: Muscarinic M1 receptors modulate endotoxemia-induced loss of synaptic plasticity
Source: Acta Neuropathol Commun. 2015 Nov 4;3:67. doi: 10.1186/s40478-015-0245-8 (PMC4632469; doi:10.1186/s40478-015-0245-8)
Supplement: Additional file 3: — Results summary. Tables show measured values from all experiments in this study. Data is presented as mean ± SEM. (PDF 31 kb) [file 40478_2015_245_MOESM3_ESM.pdf]

## Supplementary Table 6

| results sheet for Figure 2 | | | | | | | | |
|---|---|---|---|---|---|---|---|---|
| mean ± SEM | **control (n = 15 cells / 11 rats)** | **LPS (n = 10 cells / 7 rats)** | **control + apamin (n = 6 cells / 4 rats)** | **LPS + apamin (n = 8 cells / 5 rats)** | **control + physo (n = 6 cells / 4 rats)** | **LPS + physo (n = 5 cells / 5 rats)** | **control + TBPB (n = 7 cells / 3 rats)** | **LPS + TBPB (n = 7 cells / 5 rats)** |
| **number of action potentials** | 11.0 ± 1.3 | 9.8 ± 1.1 | 9.8 ± 1.5 | 13.5 ± 2.1 | 15.7 ± 1.8 | 16.4 ± 1.7 | 18.4 ± 2.5 | 13.2 ± 1.0 |
| **resting membrane potential (mV)** | -56.0 ± 1.7 | -55.8 ± 1.2 | -60.0 ± 1.2 | -59.6 ± 1.5 | -55.7 ± 1.4 | -51.8 ± 1.8 | -59.3 ± 0.9 | -58.0 ± 1.4 |
| **paired pulse ratio with 50 ms IPI** | 1.40 ± 0.08 | 1.34 ± 0.06 | 1.40 ± 0.07 | 1.53 ± 0.07 | 1.37 ± 0.08 | 1.48 ± 0.08 | 1.50 ± 0.06 | 1.50 ± 0.09 |
| **paired pulse ratio with 100 ms IPI** | 1.17 ± 0.08 | 1.20 ± 0.06 | 1.24 ± 0.09 | 1.25 ± 0.08 | 1.31 ± 0.05 | 1.32 ± 0.05 | 1.37 ± 0.05 | 1.33 ± 0.07 |

| results sheet for Figure 3 | | | | | | | | |
|---|---|---|---|---|---|---|---|---|
| mean ± SEM | **control (n = 14 cells / 11 rats)** | **LPS (n = 8 cells / 7 rats)** | **control + apamin (n = 6 cells / 4 rats)** | **LPS + apamin (n = 8 cells / 5 rats)** | **control + physo (n = 6 cells / 4 rats)** | **LPS + physo (n = 5 cells / 5 rats)** | **control + TBPB (n = 7 cells / 3 rats)** | **LPS + TBPB (n = 6 cells / 4 rats)** |
| **AHP amplitude after single AP (mV)** | -10.8 ± 0.5 | -13.8 ± 0.9 | -7.8 ± 0.9 | -7.4 ± 0.7 | -9.2 ± 0.3 | -10.5 ± 0.7 | -9.3 ± 0.5 | -8.4 ± 1.1 |
| **hyperpolarization amplitude after AP burst** | -7.0 ± 0.7 | 10.1 ± 0.5 | -5.4 ± 0.4 | -4.4 ± 0.6 | -4.1 ± 0.4 | -7.9 ± 1.1 | -4.4 ± 0.5 | -5.1 ± 0.7 |

| results sheet for Figure 4 | | | | | | | | |
|---|---|---|---|---|---|---|---|---|
| mean ± SEM | **control (n = 15 cells / 11 rats)** | **LPS (n = 10 cells / 7 rats)** | **control + apamin (n = 6 cells / 4 rats)** | **LPS + apamin (n = 8 cells / 5 rats)** | **control + physo (n = 6 cells / 4 rats)** | **LPS + physo (n = 8 cells / 8 rats)** | **control + TBPB (n = 7 cells / 3 rats)** | **LPS + TBPB (n = 7 cells / 5 rats)** |
| **normalized EPSC amplitude** | 1.78 ± 0.05 | 1.08 ± 0.02 | 1.9 ± 0.1 | 1.77 ± 0.06 | 1.55 ± 0.03 | 1.31 ± 0.02 | 1.64 ± 0.02 | 1.40 ± 0.03 |

| results sheet for Figure 5 | | | | | | | | |
|---|---|---|---|---|---|---|---|---|
| mean ± SEM | **control (n = 8 cells / 5 rats)** | **LPS (n = 10 cells / 7 rats)** | **control + apamin (n = 6 cells / 4 rats)** | **LPS + apamin (n = 8 cells / 5 rats)** | **control + physo (n = 6 cells / 4 rats)** | **LPS + physo (n = 6 cells / 6 rats)** | **control + TBPB (n = 7 cells / 3 rats)** | **LPS + TBPB (n = 7 cells / 5 rats)** |
| **number of spikes** | 7.6 ± 0.9 | 7.9 ± 0.5 | 9.0 ± 1.3 | 10.3 ± 1.3 | 16.5 ± 0.9 | 9.7 ± 1.5 | 15.1 ± 1.3 | 10.3 ± 1.3 |
| **AUC (mVms x $10^{-3}$)** | 25442 ± 3135 | 22102 ± 1392 | 19058 ± 2148 | 32351 ± 2763 | 24617 ± 1173 | 33606 ± 2531 | 24819 ± 1193 | 28862 ± 1427 |
